# Draft Genome Sequence of the Non-Microcystin-Producing Microcystis aeruginosa Strain KLA2, Isolated from a Freshwater Reservoir in Northern California, USA

**DOI:** 10.1128/MRA.01086-19

**Published:** 2020-01-16

**Authors:** Pia H. Moisander, Mari Ochiai

**Affiliations:** a Department of Biology, University of Massachusetts Dartmouth, North Dartmouth, Massachusetts, USA; b Ocean Sciences Department, University of California Santa Cruz, Santa Cruz, California, USA; c Center for Marine Environmental Studies, Ehime University, Matsuyama, Japan; University of California, Riverside

## Abstract

We report a draft genome sequence for Microcystis aeruginosa KLA2. The total draft genome size is 5,213,465 bp with a GC content of 42.5%. The genome does not have genes indicative of microcystin production but does contain genes indicative of production of several other secondary metabolites.

## ANNOUNCEMENT

Strain KLA2 of the unicellular colony- and bloom-forming cyanobacterium Microcystis aeruginosa was isolated from the Copco Reservoir on the Klamath River, California (41.984N, −122.325W), in July 2007. Water was spread on BG11 agar (1) with a reduced nitrate content of 1.76 mM (BG11N−), and plates were grown under a 15-h/9-h light/dark cycle. Picked colonies were subsequently grown in liquid BG11N−. The strain was considered a non-microcystin producer, as the *mcyA* gene (encoding part of the microcystin gene cluster) did not amplify (2) and microcystin was never detected in multiple tests conducted during the years before and after genome sequencing (enzyme-linked immunosorbent assay [ELISA] kit; Envirologix, Portland, ME, USA).

Strain KLA2 was first purified using fluorescence-activated cell sorting (BD Influx; Beckton Dickinson, USA). To prepare the culture for sequencing, cells were agitated briefly in a bead beater in tubes without beads to break up colonies and to collapse gas vacuoles. Cells were then filtered through a 10-μm filter to reduce aggregation. Cells (10,000 total) were then flow-sorted to nuclease-free water and frozen. The sorted cells directly underwent whole-genome amplification as described previously (REPLI-g midikit; Qiagen, USA) (3). Library preparation and sequencing were conducted at the University of California Santa Cruz Genome Sequencing Center on the Roche 454 Titanium platform (∼450-bp reads), and a subsequent SOLiD mate pair run was conducted to improve the assembly (sequencing was conducted in 2009 and 2010, respectively). The 659,000 454 reads (246 Mb total) were assembled with Newbler (454 Life Sciences, Branford, CT, USA), producing 810 contigs (497 contigs of >500 bp). The SOLiD mate pair sequencing yielded 76 million reads (2 × 50 bp per read, 2- to 3-kb insert size); these reads were mapped to the 454 contigs using LifeScope 2.5.1 (Thermo Fisher) with default parameters (Life Technologies Bioinformatics Service). PCR duplicates likely resulting from library generation were filtered out during the alignment, with only the primary alignment with mapping quality of 10 or greater considered. The mapping resulted in 15 million reads (7.5 million mate pairs). Application of MIPS Scaffolder 0.6 (4) resulted in 251 scaffolds of >500 bp with a total length of 5,213,465 bp, an *N*_50_ value of 30,960 bp (5), and a GC content of 42.5%. For scaffolding, the inferred median insert size (2,167 ± 581 bp [interquartile range, 1,586 to 2,748 bp]) determined from the data was used for the mate pairs that mapped to different contigs.

Annotation was conducted with the Prokaryotic Genome Annotation Pipeline (PGAP) (6) on the version submitted to NCBI. The annotation indicated 5,215 and 4,634 total and protein-coding genes, respectively; 1 each of 5S, 16S, and 23S rRNA; and 41 tRNAs. A comparative annotation using Rapid Annotation of microbial genomes using Subsystems Technology (RAST) 2.0 (7) identified 5,841 coding sequences and 263 subsystems. Based on both annotations, genes homologous to nitrate and urea ABC transporters and urease alpha-, beta-, and gamma-units were present, suggesting the capacity for using these nitrogen sources. Both annotations also identified genes homologous for gas vesicle production, suggesting a capacity for surface bloom formation.

A total of 9 Cas gene clusters and 35 CRISPR spacer regions with up to 8 CRISPR spacer repeats per region were detected with the CRISPRCasFinder (8). The KLA2 draft genome sequence has 95.96 and 97.12% average nucleotide identity (two-way) (9) with the genomes of *M. aeruginosa* NIES-843 (10) and PCC 7806 (11), respectively, which both contain the microcystin gene cluster. Microcystin genes were not found in KLA2. Several other entire or partial secondary metabolite biosynthetic gene clusters were found using antiSMASH 5.0 with default parameters (Table 1) (12). The potential for secondary metabolite production in a non-microcystin-producing strain should contribute to investigations of the evolution and roles of secondary metabolites in cyanobacteria (13) and their potential impacts on aquatic ecosystems (14).

**TABLE 1:** Secondary metabolite biosynthetic gene clusters in the *M. aeruginosa* KLA2 draft genome sequence

Product type^*a*^	Most similar known cluster	Similarity (%)
Cyanobactin	Piricyclamide	58^*b*^
Cyanobactin	Piricyclamide	25^*b*^
Cyanobactin, LAP	Aeruginosamide	100
Lanthipeptide, bacteriocin	NA^*c*^	NA^*b*^
Bacteriocin	Yersiniabactin	2^*b*^
Microviridin	Microviridin B	100
hglE-KS	NA	NA^*b*^
T1PKS	Cylindrocyclophane	1^*b*^
T1PKS	Puwainaphycins	30^*b*^
T1PKS	Bartoloside 2/bartoloside 3/bartoloside 4	27^*b*^
T3PKS, T1PKS	Merocyclophane C/merocyclophane D	44^*b*^
NRPS	Aeruginosin	85^*b*^
NRPS	Micropeptin	100^*b*^
NRPS, beta-lactone	Anabaenopeptin	100^*b*^
Resorcinol	Bartoloside 2/bartoloside 3/bartoloside 4	45
Terpene	NA	NA^*b*^
Terpene	NA	NA
Terpene	NA	NA^*b*^

a LAP, linear azol(in)e-containing peptides; hglE-KS, heterocyst glycolipid synthase-like PKS; T1PKS, type 1 polyketide synthase; NRPS, nonribosomal peptide synthetase cluster.

b Region of the gene cluster was on the contig edge, which may have reduced the similarity values of <100%.

c NA, not available.

### Data availability.

This whole-genome shotgun project has been deposited at DDBJ/ENA/GenBank under the accession number VTRR00000000. The version described in this paper is version VTRR01000000. The study has been deposited under BioProject number PRJNA561215 and BioSample number SAMN12611847. The raw sequence reads are in the Sequence Read Archive under the numbers SRR10053317 and SRR10079464.
